# Challenges in identifying mRNA transcript starts and ends from long-read sequencing data

**DOI:** 10.1101/gr.279559.124

**Published:** 2024-11

**Authors:** Ezequiel Calvo-Roitberg, Rachel F. Daniels, Athma A. Pai

**Affiliations:** RNA Therapeutics Institute, University of Massachusetts Chan Medical School, Worcester, Massachusetts 01605, USA

## Abstract

Long-read sequencing (LRS) technologies have the potential to revolutionize scientific discoveries in RNA biology through the comprehensive identification and quantification of full-length mRNA isoforms. Despite great promise, challenges remain in the widespread implementation of LRS technologies for RNA-based applications, including concerns about low coverage, high sequencing error, and robust computational pipelines. Although much focus has been placed on defining mRNA exon composition and structure with LRS data, less careful characterization has been done of the ability to assess the terminal ends of isoforms, specifically, transcription start and end sites. Such characterization is crucial for completely delineating full mRNA molecules and regulatory consequences. However, there are substantial inconsistencies in both start and end coordinates of LRS reads spanning a gene, such that LRS reads often fail to accurately recapitulate annotated or empirically derived terminal ends of mRNA molecules. Here, we describe the specific challenges of identifying and quantifying mRNA terminal ends with LRS technologies and how these issues influence biological interpretations of LRS data. We then review recent experimental and computational advances designed to alleviate these problems, with ideal use cases for each approach. Finally, we outline anticipated developments and necessary improvements for the characterization of terminal ends from LRS data.

The development of long-read sequencing (LRS) technologies has heralded a new era that advances the ability to interrogate RNA at single-molecule resolution, characterize the full composition of exons on individual mRNAs (Tilgner et al. 2014; Tian et al. 2021; Wright et al. 2022b), and determine the connections between protein-coding and untranslated regions (UTRs) that regulate mRNA functions and expression. Furthermore, the quantification of mRNA isoforms using LRS can provide data on their relative usage across cell types (Au et al. 2013; Sharon et al. 2013; Byrne et al. 2017; Gupta et al. 2018), cellular contexts (Wright et al. 2022b), and disease conditions (Rausch et al. 2023) to inform research related to disease mechanisms and public health (Maestri et al. 2020; Beyter et al. 2021). Thus, LRS has the potential to enable a functional understanding of how and why specific full-length mRNA isoforms are expressed. LRS technologies have been applied for the study of many organisms, spanning from the description of very long bacterial operons (Grünberger et al. 2022) to the uncovering of the full diversity of human mRNAs.

However, there remain challenges in the widespread implementation of LRS for RNA-based applications. Although much focus has been placed on the advantages and challenges of LRS in defining exon composition and structure in mRNA isoforms (Tang et al. 2020; Chen et al. 2023; Pardo-Palacios et al. 2024b), the ability to identify and quantify the terminal ends of mRNA molecules—specifically, the transcript start and end (polyadenylation) sites (TSSs and PASs, respectively)—has been less carefully investigated. Such characterization is crucial for completely delineating the full mRNA molecule and regulatory functions encoded in UTRs.

Herein, we discuss the importance of characterizing mRNA terminal ends with LRS and, through a systematic re-analysis of publicly available LRS data sets, illustrate the limitations of terminal end identification and quantification. We outline experimental and/or computational issues that may underlie these biases and review current approaches that aim to alleviate them. Finally, we provide a long-term perspective on necessary advances to improve LRS in RNA-based applications.

## mRNA terminal ends are regions of great biological importance

**Figure 1.**
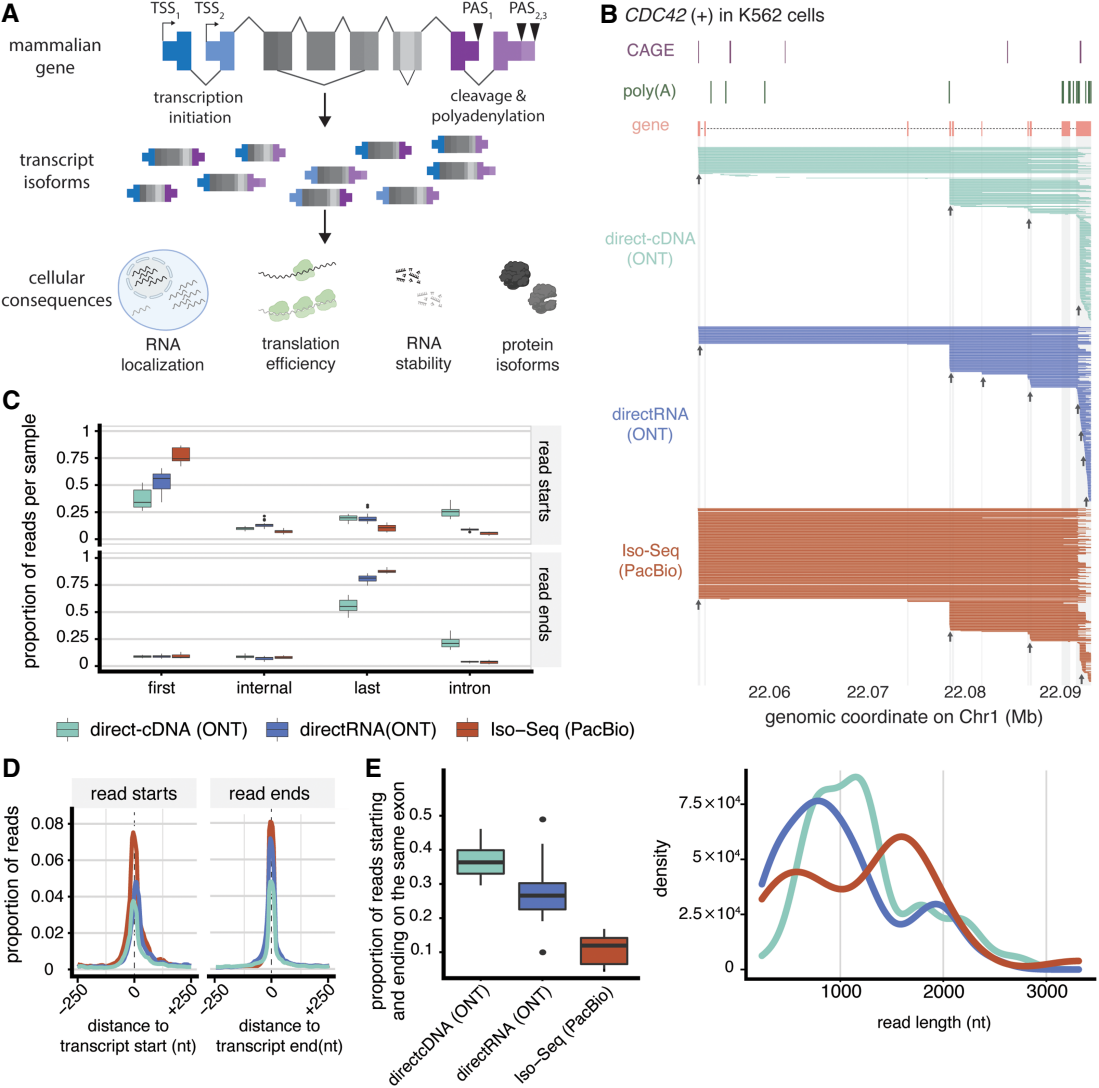
LRS reads show large variability in start and end coordinates. (*A*) Mammalian genes can have multiple alternative transcript starts (TSSs) and ends (PASs), leading to different isoforms expressed from the same gene with variable cellular consequences. (*B*) Representative example of long-read RNA sequencing reads for *CDC42* in K562 cells. To aid visualization, 280 randomly sampled reads from each sequencing technology are shown. At the *top* are annotated features of *CDC42*, including CAGE peaks (violet), poly(A) peaks (dark green), and annotated exons (orange). At the *bottom* are read coverage plots, in which each horizontal line represents the span between the first and last coordinates of a read, for the ONT direct-cDNA (teal) (Chen et al. 2021), ONT directRNA (blue) (Chen et al. 2021), and PacBio Iso-Seq (red) (Luo et al. 2020) data sets (Supplemental Methods; Supplemental Table S1). Arrows mark TSS clusters. The *bottom* panel shows the distribution of read length across sequencing technologies for *CDC42*. (*C*) Distributions of the proportion of reads that start (*top*) or end (*bottom*) in HITindex-classified (Fiszbein et al. 2022) first, internal, or last exons or introns across from three LRS technologies, using data from the A549, Hct116, HepG2, K562, and MCF-7 cell lines. (*D*) The distribution of read starts and ends around annotated transcription start (*left*) or end (*right*) sites across three LRS technologies. The *y*-axis represents the proportion of reads per sample, calculated using a sliding window of 0.01 kb around the feature. (*E*) Distributions of the proportion of single-exon reads that start and end within the same exonic feature across three LRS technologies. Note that *B*–*E* use published data sets, and the metrics presented here may change when estimated with other data sets or protocols.

The biogenesis of eukaryotic mRNA molecules involves multiple molecular mechanisms, each of which can be alternatively regulated. Messenger RNAs are synthesized by RNA Polymerase II (RNAPII), which is recruited to TSS (Juven-Gershon et al. 2008). As nascent RNAs emerge from the RNAPII exit channel, a seven-methyl-guanosine cap is added to protect the 5′ end (Galloway and Cowling 2019). Splice sites on the pre-mRNA are recognized by spliceosomal complexes to excise introns, often as the molecule is actively being elongated (Carrocci and Neugebauer 2019; Zhang et al. 2021). Finally, recognition of a polyadenylation signal (PAS) by the RNA cleavage complex initiates cleavage and polyadenylation (addition of a poly(A) tail) to form the 3′ end of the mRNA molecule (Tian and Manley 2016). Each of these events—transcription initiation, pre-mRNA splicing, and 3′ end cleavage and polyadenylation—can be alternatively regulated to create a multitude of mRNA isoforms from a single gene region (Fig. 1A). Importantly, splicing occurs at specific splice sites on an mRNA (delineated by strong sequence motifs), but there is more stochasticity in the choice of specific TSS and PAS sites, where these sites are often thought to occur in clusters rather than single positions (Geisberg et al. 2022).

Although much attention has been given to alternative splicing of internal exons, alternative regulation at the 5′ and 3′ ends of the transcript contributes just as much, if not more, to isoform diversity (Shabalina et al. 2014; Reyes and Huber 2018). There are thought to be more than 37,000 and more than 85,000 known alternative TSSs and PASs, respectively, in the human genome (Wang et al. 2018; Herrmann et al. 2019; Moore et al. 2022), with an average of five and four alternative TSSs and PASs per gene, respectively (Wang et al. 2018; Abugessaisa et al. 2021). Alternative terminal ends can expand or truncate protein sequences, but more frequently, they do not change coding regions and instead determine the composition of the 5′ and 3′ UTRs of an mRNA molecule. UTRs play important roles in post-transcriptional regulation by containing sequence features that influence mRNA stability, localization, and translation efficiency (for review, see Di Giammartino et al. 2011). TSS usage delineates the 5′ UTR, which is involved in ribosome loading and translation efficiency and can influence the expressed open reading frame (Leppek et al. 2018).

At the other end, alternative PASs dictate the 3′ UTR, which is involved in the regulation of mRNA stability and localization and can also lead to protein truncation (Di Giammartino et al. 2011; Lianoglou et al. 2013; Taliaferro et al. 2016; Alfonso-Gonzalez and Hilgers 2024). Furthermore, lack of proper 3′ end cleavage induced by cellular stresses or other perturbations can cause transcriptional readthrough, which alters the 3′ ends of mRNAs (for reviews, see Vilborg and Steitz 2017; Rosa-Mercado and Steitz 2022). Thus, through a mix of effects on protein composition, biogenesis, and levels, alternative terminal ends may significantly impact protein diversity (Wang et al. 2018; Abugessaisa et al. 2021; Carrion et al. 2023)

Although the regulation of alternative TSSs, splicing, or PASs on an mRNA have all historically been thought to occur independently, increasing evidence suggests extensive coordination to regulate the expression of full-length isoforms (Alfonso-Gonzalez and Hilgers 2024). For instance, recent studies have identified coupling between alternative TSSs and splicing (Anvar et al. 2018; Fiszbein et al. 2019; Alfonso-Gonzalez et al. 2023), splicing and alternative TESs (Anvar et al. 2018; Alfonso-Gonzalez et al. 2023), and alternative TSS and PAS mechanisms (Anvar et al. 2018; Alfonso-Gonzalez et al. 2023; Calvo-Roitberg et al. 2024). Furthermore, alternative splice sites or PASs may compete for usage, and splicing or 3′ polyadenylation decisions may influence each other within an mRNA molecule (Coulon et al. 2014; Reimer et al. 2021; Choquet et al. 2023; Zhang et al. 2023). The coordination between these processes highlights the need to move beyond characterization of individual exons toward studying alternative RNA processing events in the isoform context. Therefore, LRS provides an attractive approach for studying full-length RNA molecules.

## Challenges of terminal end identification with long-read RNA sequencing

Short-read sequencing (SRS) technologies are the gold standard for high-throughput transcriptome profiling (Wang et al. 2009). RNA-sequencing (RNA-seq) has been used to investigate gene expression (The ENCODE Project Consortium 2012), canonical and alternative mRNA splicing (Wright et al. 2022a), noncoding RNAs, and post-transcriptional modifications (Jia et al. 2020) among many other applications. However, SRS technologies are usually limited by their inability to sequence more than 100–300 contiguous nucleotides (nt). Although SRS offers robust and accurate solutions for detecting and characterizing local features within the sequenced fragment, these data cannot inform exon connectivity across a full isoform. The average transcript length in most species is much longer than the average SRS read length (i.e., human transcripts are ∼2000 nt on average), leading to computational approaches to infer full transcript levels and compositions (Trapnell et al. 2010; Bray et al. 2016; Patro et al. 2017; Hölzer and Marz 2019). These methods often anchor their analyses on known sequences or databases of previously observed transcripts. Therefore, although they succeed at gene expression quantification (in which gene boundaries are well identified) and transcript quantification of known isoform structures, these approaches cannot accurately identify and quantify new isoforms that involve previously uncharacterized connections between exons (Conesa et al. 2016). This is especially problematic for the identification and quantification of exons at the terminal ends of transcripts because there are no common features to define reads from these regions (Qin et al. 2018; Cass and Xiao 2019; Fiszbein et al. 2022). The challenge of characterizing mRNA terminal ends in SRS data is also magnified by fragmentation and size selection steps during library preparation, which lead to an “edge-effect” in which there is a depletion of reads from the ends of transcripts (Carralot et al. 2012; Birol et al. 2015).

LRS technologies promise the ability to directly sequence full-length mRNA molecules without fragmentation and subsequent computational inference of the relationships between individual exons. The LRS technology space is currently dominated by Pacific Biosciences (PacBio; https://www.pacb.com/products-and-services/applications/rna-sequencing/ [accessed April 25, 2024]) and Oxford Nanopore Technologies (ONT) (Box 1). PacBio utilizes single-molecule real-time (SMRT) sequencing, in which circularized cDNAs are sequenced by detecting pulse waves resulting from the excitation of fluorescent dNTPs during the synthesis of a complementary strand (Iizuka et al. 2022). In contrast, ONT nanopore motors directly pull molecular fragments (double-stranded cDNA or “native” RNA) through a pore as a constant electrical current is applied. Each nucleotide (or modified nucleotide) within the stretch of nucleotides in the nanopore results in a characteristic voltage signal, allowing the sequencing of a molecule without requiring a synthesis step (Pugh 2023).

Box 1. **Overview of current long-read RNA sequencing technologies**

Two main companies, Pacific Biosciences (PacBio) and Oxford Nanopore Technologies (ONT) offer commercial solutions for long-read RNA sequencing.

***PacBio*.** In this technology, a single-stranded circularized cDNA molecule is generated by ligating hairpin adaptors to double-stranded cDNA (SMRTbell library preparation) and applied to small wells embedded in proprietary SMRT cells. Each well contains a single immobilized φ29 DNA polymerase molecule and a pool of phospholinked (four-color) fluorescent dNTPs. Single-molecule sequencing is performed by reading fluorescent signals produced in real-time through base-pairing events between nucleotides in the template strand with complementary dNTPs. Correct base-pairing results in a longer dwell time in the polymerase, corresponding to a characteristic pulse wave detected by the instrument during this synthesis phase. PacBio's high-fidelity long-read (HiFi) technology uses an enzyme with processivity greater than the insert length; thus, the circularized template is sequenced multiple times to obtain circular consensus reads with high accuracy when combining information across iterative sequencing instances.

***ONT.*** In this technology, nanopores are embedded in an electro-resistant membrane and suspended between two chambers with ionic solutions, through which an electric current is passed. Single- or double-stranded molecules with ligated adaptors are bound to an enzyme at the mouth of the pore that “unzips” the strands as applied electric potential causes a single strand to be electrokinetically pulled through the pore. Stretches of 10–15 nt travel through the pore, causing a temporary blockage of the current, with electrical signatures that are specific to each nucleotide (or modified nucleotide). The RNA applications of ONT include direct RNA sequencing (which preserves base modification information lost during traditional cDNA synthesis), in which the RNA molecules are either ligated directly to the RNA molecule or subjected to “splinting” using single-strand reverse transcription to generate a hybrid DNA-RNA molecule that is more efficiently processed by the pore to read the RNA strand of this molecule (Garalde et al. 2018). ONT can also be used to sequence cDNA molecules (Grünberger et al. 2022; Wongsurawat et al. 2022).

***LRS library preparation approaches.*** RNA isolation and purification are shared between all LRS sequencing methods. Similarly, library construction involving cDNA synthesis is fundamentally similar between ONT and PacBio. Reverse transcription is initiated by primers that enrich for specific target RNA populations, commonly either oligo(dT) or random hexamers, depending on the specific application. Oligo(dT) primers enrich for mature RNAs transcribed by RNAPII by annealing to the polyadenylated tail present at the 3′ end of most eukaryotic mRNA molecules. Random hexamers anneal to various sites along the RNA molecules, allowing for reverse transcription of the entire RNA population starting from different sites.

The reverse transcriptase enzyme then uses the RNA molecule as a template to synthesize a cDNA strand by extending the primer to synthesize the first strand of cDNA (fs-cDNA). Long-read library preparation methods commonly use template-switching reverse transcriptase enzymes, which add a nontemplated “CCC” triplet at the 5′ end of the fs-cDNA. A template-switching oligo (TSO) with a complementary “GGG” can then bind the “CCC” and allow the reverse transcriptase enzyme to immediately begin synthesizing a double-stranded second-strand cDNA (ss-cDNA) using the first cDNA strand as a template.

Following cDNA synthesis, technology-specific adapters are ligated to the completed molecules. For ONT, these adapters include a sequencing adapter attached to a motor protein that drives the translocation of the cDNA molecule through the pore. The adapter additionally concentrates DNA substrates at the membrane surface near the nanopore to increase DNA capture. The SMRTbell adapters in PacBio technology include two adapters, each of which includes a double-stranded stem and a single-stranded hairpin loop. The two adapters may have identical (symmetric) or different (asymmetric) sequences. The ligation of these adapters to double-stranded cDNA allows the formation of a single-stranded circular DNA template that is read by the embedded polymerase.

**Box Figure 1.**
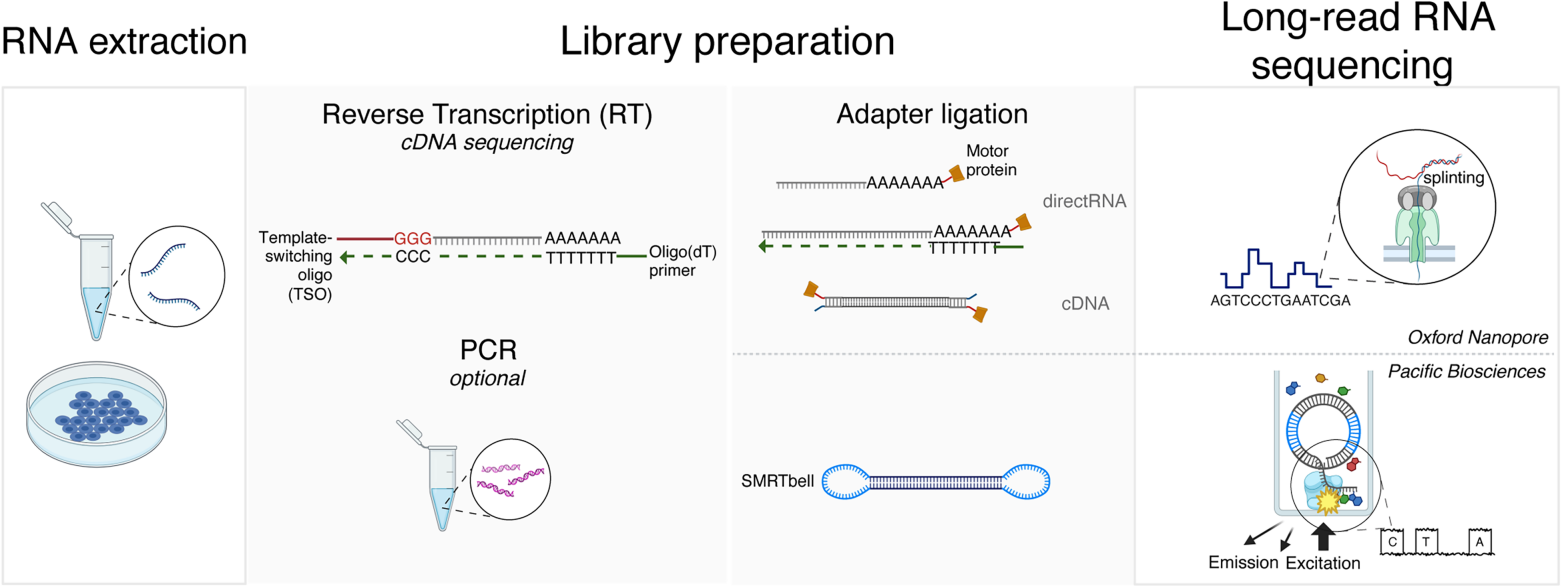
Overview of current long-read RNA sequencing technologies. Depiction of RNA preparation, library preparation, and sequencing steps for ONT and PacBio technologies. On the *left* are shared RNA extraction and library preparation steps, in which reverse transcriptase is only required for the cDNA sequencing methods (both PacBio and ONT) and PCR is required for PacBio but optional for ONT. On the *right* are depictions of the varying adapter ligation and sequencing for ONT (*top*) and PacBio (*bottom*) methods.

Although advances in LRS methods have improved accuracy and consistency in sequencing of full-length molecules in recent years, these techniques still face technical and computational hurdles, which vary according to LRS technology (Pardo-Palacios et al. 2024b). These include relatively low genomic coverage (compared with short-read technologies), which limits the annotation or quantification of lowly expressed genes or isoforms (Boldogkői et al. 2019), and persistent concerns regarding insertion, deletion, or misalignment errors, leading to problems with the accurate identification of single-nucleotide variations (SNVs) and internal splice sites (Amarasinghe et al. 2020; Cui et al. 2020; Dohm et al. 2020; Mikheenko et al. 2022). These challenges affect all applications of LRS for RNA profiling, including the characterization of mRNA terminal ends. Although analytical tools have been proposed to correct for some of these challenges when characterizing internal exons (Amarasinghe et al. 2020; Tang et al. 2020; Chen et al. 2023), it remains unclear how these and/or other technical challenges may uniquely impact terminal end characterization. Although low accuracy of LRS reads may affect the ability to identify precise mRNA 5′ and 3′ ends, which, unlike splice sites, are not delineated by common sequence elements (Birol et al. 2015; Arefeen et al. 2019; Dudnyk et al. 2024), this is not likely to be the major challenge for terminal end identification (as reviewed below).

Previous studies have observed decreased LRS read coverage toward the ends of mRNA molecules, suggesting limited power to characterize terminal ends (Wright et al. 2022b). Moreover, the read start and end positions, denoting TSSs and transcript end sites, are highly variable across reads (Ibrahim et al. 2021). This is evident when looking at aligned reads for an individual gene across LRS approaches and platforms. As a representative example, there is large variability in the number of TSSs identified across high-coverage LRS data sets for the *CDC42* gene in K562 cells (Supplemental Methods; Supplemental Code; Supplemental Table S1; Fig. 1B), with four, eight, and four start site clusters supported by at least 10 read starts that are <50 nt away from each other across the direct-cDNA, directRNA (Chen et al. 2021), and Iso-Seq (Luo et al. 2020), respectively. We observe less variability across methods in transcript end site identification, with three, four, and three end site clusters for *CDC42* in K562 cells in the direct-cDNA, directRNA, and Iso-Seq data sets, respectively. Notably, especially for read starts, many of the identified terminal positions do not overlap with the terminal coordinates of annotated *CDC42* isoforms or with empirically derived 5′ or 3′ sites (using CAGE and PAS-seq peaks, respectively). This is consistent with early reports that only 60% of PacBio read starts and ends map to annotated first and last exons, respectively (Sharon et al. 2013). Although it is very likely that the reference databases do not contain all alternative terminal sites (Tang et al. 2020; Wyman et al. 2020; Tian et al. 2021), this cannot explain the large variability between different LRS approaches. For techniques that use adapter sequences to tag the 5′ and 3′ ends of molecules during library preparation (see below), one report in the nascent data observed that as few as 7% of reads actually contain these adapters (Reimer et al. 2021). Additionally, in an approach that uses both 5′ and 3′ adapters, 80% of the 3′ ends have the correct adapter, but only as many as 32% of the 5′ ends have the correct adapter (Ibrahim et al. 2021). When molecules have the 5′ adapter, they tend to also have the 3′ adapter. Similar analyses looking for nontemplated 3′ adenosines have seen that reads without poly(A) tails often do not match known 3′ ends (Wright et al. 2022b).

More systematically, when evaluating how many read start and end positions occur in empirically derived first, internal, or last exons, we see that a substantial proportion of read starts and ends do not fall within the expected first or last exons, respectively (Fig. 1C). This is especially true for read starts in the ONT approaches, in which >50% and >40% of read starts in the direct-cDNA and directRNA libraries, respectively, do not fall within first exons. Consistently, for ONT methods, we find that a higher proportion of read starts are found within last exons. Similar trends are observed for read start and end distributions around annotated TSSs or PASs. We see that all methods have a slight shift toward read start positions downstream from annotated TSSs, with a higher density of read starts centered around the annotated TSSs for PacBio Iso-Seq data (Fig. 1D). The positioning of read ends for all sequencing techniques is much more tightly distributed around annotated PAS, consistent with previous observations that although the 3′ ends of LRS RNA-seq data tend to fall within ∼5 nt of an annotated transcript end, there is a wider distribution (∼15–100 nt) of the 5′ end positioning around annotated TSSs (Sharon et al. 2013). A final surprising observation is that many LRS reads appear to be derived from a single exon. For instance, 36%, 27%, and 10% of reads across the direct-cDNA, directRNA, and Iso-Seq data sets, respectively, exhibited no splicing and were fully contained within the 3′ most exon of *CDC42*, despite no annotated single-exon isoforms. More broadly, >25% of reads generated by ONT methods can be classified as single-exon reads (Fig. 1E). Given the prevalence of these types of reads, recent computational approaches have included a “monoexon” category that classifies putative transcripts assembled from these reads as incomplete (Pardo-Palacios et al. 2024a). Although it is not possible to rule out that LRS approaches may be especially sensitive to uncover extremely widespread stochasticity in terminal site precision and novel isoform usage driven by alternative terminal ends, it is more likely that LRS reads often have spurious terminal ends.

Altogether, these observations suggest widespread 5′ truncation in LRS reads, particularly in those from ONT approaches (Mikheenko et al. 2022). A relatively low proportion of read terminal ends across LRS approaches fall in introns; thus, these reads likely represent mature mRNA molecules rather than DNA contamination, and 5′ truncation may occur during library preparation, sequencing, or processing of the data. The presence of truncation is also supported by the relatively shorter reads generated by ONT directRNA and direct-cDNA approaches (Pardo-Palacios et al. 2024b). However, it is still difficult to disambiguate whether these terminal end inconsistencies occur owing to technical or biological causes. One option to better understand these observations is to leverage biological expectations, in which single-exon genes without any alternative isoform regulation should be less likely to have variability in 5′ end read positions. Incomplete LRS coverage is positively correlated with the length of single-exon genes, such that longer single-exon genes show more variability in 5′ read position (Workman et al. 2019). Similarly, transcripts >2 kb in length tend to have lower coverage in ONT directRNA sequencing data sets (Soneson et al. 2019; Workman et al. 2019). To probe technical biases, Mikheenko et al. (2022) devised a strategy to use unique molecular identifiers (UMIs) to tag individual molecules during reverse transcription (RT) and sequence the same cDNA library with PacBio and ONT approaches. This allowed the direct assessment of terminal end identification across platforms. After stringent filtering for reads within 100 nt of an annotated poly(A) site or CAGE peak and at least one read within a set of reads sharing a UMI being assigned directly to a TSS or PAS, the authors observed that 95% of PacBio and ONT reads had the same 3′ end, but only 87% had the same 5′ end. The representation of known and novel isoforms in LRS data is influenced by both sample preparation and sequencing technology. For example, technologies with limited read length may fail to capture the full diversity of transcript ends, whereas low read depth can hinder the detection of low-abundance isoforms (Pardo-Palacios et al. 2024b). As LRS technologies continue to advance, it is important to balance these considerations when selecting an appropriate method.

### mRNA terminal ends are affected by LRS experimental biases

Recent observations indicate that LRS reads exhibit truncation at the 5′ and, to a lesser extent, the 3′ ends of mRNA transcripts (Depledge et al. 2019; Workman et al. 2019; Ibrahim et al. 2021; Mikheenko et al. 2022). Thus, there is a general shift in the distribution of LRS read lengths toward shorter lengths relative to the presumed true distribution of mRNA lengths (Mikheenko et al. 2022). Many experimental steps could introduce biases in LRS read lengths and, thus, lead to incorrect identification and quantification of mRNA terminal ends. Below, we explore how general RNA handling and extraction practices, library preparation steps, and inherent sequencing biases can contribute to these effects.

**Figure 2.**
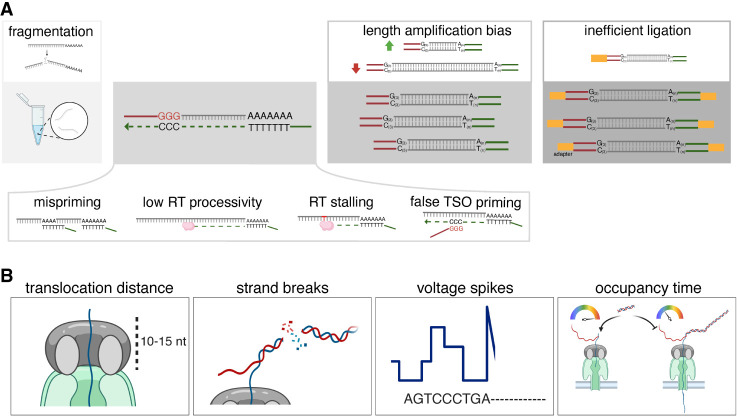
Technical biases affecting the accuracy of terminal end sequences. (*A*) Multiple events during RNA handling and library preparation can introduce biases that influence the accuracy of terminal ends in LRS reads. (*B*) Nanopores present unique inherent hardware and software challenges that affect accurate sequencing, especially at the terminal ends of molecules.

Several aspects of RNA handling before and during library preparation can significantly impact the representation of full-length mRNA molecules in LRS data (Fig. 2A). Common RNA extraction and clean-up procedures using either purification columns or nucleic acid affinity beads lead to a bias in the size of RNA molecules retained, as these procedures deplete very short (<200 nt) molecules or selectively enrich for a particular length distribution depending on the ratio of nucleic acids to affinity beads (Shi et al. 2021). These biases persist throughout library preparation, affecting the length distribution of cDNA molecules during the clean-up and downstream steps (Byrne et al. 2019). Furthermore, given the fragility of RNA molecules, they are highly subject to random fragmentation during any molecular manipulations (Fig. 2A). This fragmentation can be caused by mechanical (e.g., shearing during pipetting), enzymatic (e.g., nuclease contamination), or chemical (e.g., guanidinium) factors, all leading to systematic shortening of RNA or cDNA molecules (Byrne et al. 2019; Amarasinghe et al. 2020). Because random fragmentation should equally affect both ends of RNA or cDNA molecules, the increased prevalence of 5′ truncation in LRS reads suggests that other causes might be the primary drivers of biased representation of mRNA terminal ends. For instance, in methods that use the 3′ end to synthesize cDNA or initiate sequencing, molecules truncated near the 3′ end would be underrepresented in the final sequencing data set, thus accentuating the 5′ truncation bias.

#### Limitations during RT

Most LRS protocols involve RT at some stage during library preparation. However, RT enzymes are inherently limited in their ability to successfully generate full-length cDNA reflective of the full-length mRNA molecule (Fig. 2A; Minshall and Git 2020; Verwilt et al. 2023). In particular, the processivity of RT enzymes (the number of nucleotides an RT enzyme can incorporate into a cDNA strand before dissociating from the RNA template) is often not sufficient to reach the true 5′ end of an mRNA molecule. RT processivity highly varies, and typical RTs can synthesize 5–7 kb amplicons under standard conditions (Martín-Alonso et al. 2021), but more persistent enzymes have been developed recently (e.g., Marathon [Zhao et al. 2018] and TGIRT [Mohr et al. 2013]) (for review, see Martín-Alonso et al. 2021; Oscorbin and Filipenko 2021). Limited processivity and/or stalling of the enzyme can lead to premature termination of cDNA synthesis and truncated molecules with 5′ ends that do not represent the true mRNA 5′ end (Wyman et al. 2020).

RT processivity can be impacted by many factors, including sequence (i.e., stretches of GC-rich regions) (Minshall and Git 2020), RNA secondary structure (Verwilt et al. 2023), and RNA modifications (Kellner et al. 2010). Mispriming can also occur during RT, leading to the representation of erroneous 3′ ends. Specifically, oligo(dT) primers, which are designed to anneal to the poly(A) tail, can bind to and prime off of internal stretches of adenines within the mRNA, resulting in cDNA 3′ ends that fall upstream of the true poly(A) site (Nam et al. 2002; Balázs et al. 2019; Wyman et al. 2020). This issue can be mitigated by the ligation of a 3′ adapter ligation step before RT (see below). At a lower frequency, mispriming can also occur during template-switching RT, whereby template-switching oligos can be mispositioned owing to an internal CCC sequence, leading to erroneous 5′ ends with an adapter that suggests complete RT (Cocquet et al. 2006; Tang et al. 2013; Moldován et al. 2018). In addition, the sequence or chemical modification at the 5′ end of mRNAs may affect the efficiency of template switching (Wulf et al. 2019, 2022). However, two pieces of evidence support the idea that RT processivity or mispriming only contributes in part to the inaccurate representation of terminal sites. First, the average LRS read length is slightly shorter than the average molecule length of cDNA after RT (Soares et al. 2022; Shiau et al. 2023). Second, there is a higher prevalence of 5′ truncations in directRNA ONT sequencing data, which have an optional RT step relative to cDNA sequencing methods, but there is no observable difference in the prevalence of 3′ truncations (Pardo-Palacios et al. 2024b).

#### Limitations during library preparation

The process of converting RNA molecules to cDNA molecules involves many molecular steps and nucleic acid clean-ups during which fragmentation and size selection could impact the length of retained RNA or cDNA molecules. The largest shift in length, however, is likely introduced by polymerase chain reaction (PCR) steps at the end of library preparation, which favors the amplification of shorter fragments (Fig. 2A; Shagin et al. 1999; Parekh et al. 2016). This bias should again equally affect misrepresentation of both the 5′ and 3′ ends. However, because PCR involves priming off adapter sequences that are either added to RNA molecules during RT or ligated to cDNA molecules, any biases in the positioning of these adapters will be propagated during PCR. Further, any molecule that does not include an adapter sequence at one or both ends will fail to be amplified, which is more likely to negatively impact longer RNA molecules that were incompletely reverse-transcribed (Fig. 2A).

#### Limitations during sequencing

During both nanopore and well-based sequencing methods, shorter molecules are more likely to get sequenced because they clear the pore or pass through the polymerase more often (Fig. 2B; Baslan et al. 2021). This equally affects molecules with truncated 5′ or 3′ ends but leads to increased coverage of shorter molecules with truncated ends, propagating all the biases described above. Furthermore, DNA strands might be broken as they are translocated through the pores (particularly during the mechanical forces applied during ONT sequencing), resulting in truncation events that are more likely to occur at the 5′ end, because molecules are sequenced in the 3′-to-5′ direction (Fig. 2B; Workman et al. 2019). After 36 h of sequencing, 5% of mitochondrial RNA reads show decreased length, suggesting that strand breaks are not very abundant (Workman et al. 2019). The characteristics of nanopores may affect the accuracy of terminal ends by influencing the translocation speed at which nucleic acid molecules, both RNA or cDNA, pass through the pore. In ONT sequencing, the motor protein that facilitates molecular translation is positioned 10–15 nt away from the terminal end, resulting in the final nucleotides being pulled through the pore rapidly as they are released by the enzyme (Fig. 2B). This rapid movement limits the ability to accurately identify these final nucleotides (Workman et al. 2019; Parker et al. 2020; Ibrahim et al. 2021).

Basecalling inaccuracies during nanopore sequencing are also influenced by motor protein stalling or voltage spikes (Workman et al. 2019). Motor protein stalling occurs when the protein responsible for translocating the nucleic acid molecule through the nanopore encounters obstacles or excessive resistance, causing it to pause or terminate sequencing (White et al. 2023). Similarly, voltage spikes in the electrical signal as the RNA or cDNA molecule travels through the pore can lead to basecalling issues (Fig. 2B). These sudden fluctuations can be caused by modified nucleotides and/or background electrical noise inherent to the system and can be misinterpreted as the end of the molecule, thus terminating basecalling (Jain et al. 2022). Both stalling and voltage spikes can result in reads with truncated terminal ends, with a bias toward more 5′ truncation in direct RNA 3′-to-5′ sequencing. Although these issues are widespread, how they are generated remains unknown. In particular, it is unclear to what extent these are random events or triggered by specific molecular features.

### Experimental approaches to enrich for full-length RNA molecules

In light of the difficulties involved in natively sequencing full-length RNA molecules, many experimental approaches have been developed to tackle this challenge. These approaches broadly focus on enriching for or tagging 5′ and 3′ ends of RNA molecules to ensure full-length sequencing.

In principle, nanopore directRNA sequencing allows for full-length sequencing by bypassing terminal end or length biases created during library preparation. Additionally, directRNA sequencing can identify RNA modifications (e.g., the 5′ cap at the terminal end) (Mulroney et al. 2022; Ugolini et al. 2022) that are lost during cDNA synthesis and PCR. However, directRNA sequencing has the highest amount of 5′ truncation across LRS technologies, likely owing to issues during sequencing as outlined above. This suggests that biases generated during library preparation steps may not be the primary cause of 5′ truncation in LRS data.

#### Enrichment of longer molecules

**Figure 3.**
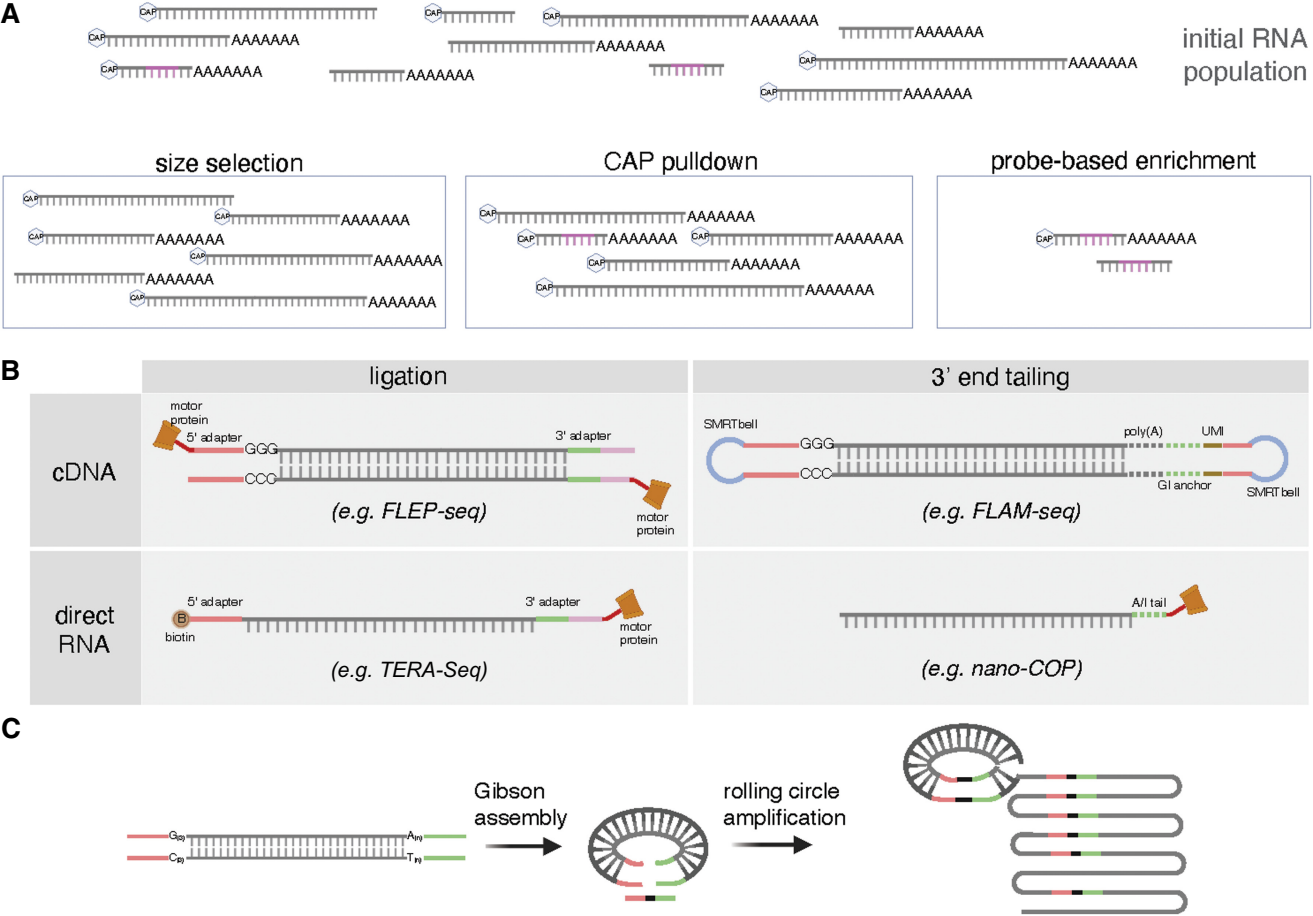
Experimental strategies to enrich for full-length RNA molecules in LRS data. (*A*) Methods that specifically enrich for 5′ ends or full-length molecules (based on size or sequence) can be used to increase the chances of sequencing full-length mRNAs. (*B*) Methods that add known sequences to the terminal ends of mRNAs by ligation (*left*) or tailing (*right*) are an unbiased way to recognize full-length molecules in both cDNA (*top*) and directRNA (*bottom*) sequencing. (*C*) Circularization of mRNAs or cDNAs during library preparation allows for multiple rounds of terminal adapter and target molecule sequencing.

As discussed above, one of the major biases in LRS is the increased probability of sequencing shorter molecules, which likely amplifies any terminal truncation effects. One strategy proposed to mitigate this issue is to enrich for longer RNA molecules before and during library preparation (Fig. 3A; Alfonso-Gonzalez et al. 2023). However, this enrichment does not entirely supersede other truncation effects created during library preparation or sequencing. The enrichment of predetermined sizes of RNA molecules may result in reads that do not represent the true distribution of molecular lengths present in the original RNA population. Thus, this process can introduce its own biases, and biological conclusions drawn from the data should be considered carefully.

#### Selective enrichment of terminal ends

To enrich for full-length RNAs, several approaches selectively isolate molecules with intact terminal end markers. RT with oligo(dT) priming targeting a poly(A) tail (as described above) represents one such strategy to selectively carry forward polyadenylated molecules. Strategies to enrich for intact 5′ ends usually involve capturing or tagging molecules with a 5′ cap (Fig. 3A). 5′ Ends can be pulled down using antibodies targeting cap modifications or a cap-binding protein (Ogami et al. 2023) or following biotinylation of the 5′ end (Jiang et al. 2019; Parker et al. 2020; Carbonell-Sala et al. 2024), with 5′ and 3′ adapter ligations that recognize the cap and poly(A) tails, respectively (e.g., CapTrap-seq) (Carbonell-Sala et al. 2024). Alternatively, the 5′ cap can be enzymatically replaced with a biotinylated RNA adapter, followed by pulldown of these cap-containing RNAs and RT primed off of a poly(A) tail to ensure full-length molecules (e.g., 5′-Cap capturing) (Jiang et al. 2019). These approaches generally generate high-quality reads, but analysis of synthetic RNAs showed fragmentation during 5′ enrichment, leading to reduced read coverage in the middle of molecules (Pardo-Palacios et al. 2024b). This may impact the characterization of terminal ends in the context of full-length isoforms and, in particular, the discovery and quantification of alternatively spliced isoforms (Maeng et al. 2023).

In principle, another enrichment-based approach to increase the sequencing of full-length isoforms involves selectively isolating transcripts from specific genes using complementary biotinylated probes (Fig. 3A; e.g., RNA capture long-read sequencing [Lagarde et al. 2017] and others [Sheynkman et al. 2020; Wang et al. 2023; Zhang et al. 2023]). This increases the coverage (number of reads) for specific genes and would probabilistically increase the number of full-length molecules sequenced. However, these libraries would still suffer from all truncation issues mentioned above without any tags or selective enrichment of terminal ends.

#### Sequence-based tagging of terminal ends

Several approaches add known adapter sequences to the 5′ and/or 3′ ends of RNA molecules to enrich for and identify full-length cDNA or RNA molecules (Fig. 3B). The simplest version is the use of a template-switching RT with a known 5′ TSO sequence, as implemented in commercial kits aiming to sequence polyadenylated RNA (e.g., TeloPrime [Balázs et al. 2019], SMARTer cDNA amplification [Cartolano et al. 2016; Legnini et al. 2019]). A more flexible approach that is agnostic to polyadenylation status involves ligation of an adapter to the 3′ end of the RNA molecule, followed by a template-switching RT with a known TSO sequence (e.g., FLEP-seq) (Herzel et al. 2018; Reimer and Neugebauer 2020; Long et al. 2021). However, because ligation is often inefficient, complementary approaches often use 3′ end guanosine and/or inosine tailing of the RNA molecule to synthesize a tail that can be used to prime a template-switching RT reaction (e.g., FLAM-seq) (Legnini et al. 2019). Approaches with similar conceptual frameworks have also been developed for directRNA nanopore sequencing. For instance, it is possible to directly ligate 5′ and/or 3′ adapters onto an RNA molecule (agnostic of polyadenylation status), which can be used to mediate ligation of the ONT sequencing adapters (e.g., TERA-Seq) (Ibrahim et al. 2021). Alternatively, 3′ end adenine or inosine tailing of the RNA molecule can be used to mediate adapter ligation directly to RNA (e.g., nano-COP) (Drexler et al. 2021).

Together, these methods all add at least one terminal tag that can be used to confirm full-length sequencing, which allows for unbiased terminal end identification and quantification without relying on independent terminal site annotations. Many of these methods can address the ONT translocation issue that prevents accurate sequencing of the final 10–15 nt by ligating longer adapter sequences. Longer adapter sequences are also helpful to prevent potential internal mispriming events at either the 5′ or 3′ end as described above. The use of longer adapters or in vitro nucleoside tailing provides a more defined target for primer binding with higher fidelity and enhances the specificity of terminal end identification, particularly at the 3′ end (Ibrahim et al. 2021). Finally, any fragmentation or length biases that occur during RT (i.e., owing to RT processivity limitations for distances longer than ∼10 kb) (Zhao et al. 2018) would be propagated during these sequence-based terminal end tagging approaches. Similarly, these methods would still suffer from all of the artifacts introduced during sequencing (e.g., strand breaks) that lead to 5′ truncation of reads, although only retaining reads with a 5′ adapter would increase the probability that reads delineate true 5′ RNA ends.

#### Sequencing of full-length molecules after circularization

Circularizing molecules after the addition of terminal adapters protects the ends from exonuclease degradation and enhances the accuracy of terminal-end sequencing by directly colocalizing 5′ and 3′ ends of molecules (Wang et al. 2020; Scarano et al. 2024). This is routinely done during PacBio library preparation with the addition of SMRTbell adapters that enable circularization and continuous sequencing of the circle (https://www.pacb.com/products-and-services/applications/rna-sequencing/ [accessed April 25, 2024]). Circularization of full-length cDNA followed by rolling circle amplification to generate multiple concaterimized sequences of the same molecule enables a similar method to be applied with ONT sequencing (e.g., R2C2) (Fig. 3C; Volden et al. 2018). These methods help to both increase read length (thus decreasing truncation effects) and reduce basecalling errors because circularization allows each molecule to be sequenced multiple times. Although these data often achieve some of the highest RNA read lengths (Pardo-Palacios et al. 2024b), these approaches are still limited by the previously mentioned biases that affect terminal end truncation.

## Computational approaches to identify and quantify full-length isoforms

Despite the development of experimental approaches to more accurately sequence full-length RNA molecules, there are still analytical hurdles to identifying and quantifying complete isoforms. Thus, many computational approaches have been developed to account and/or correct for technical biases (Table 1). Although most of these pipelines were specifically designed to analyze alternative splicing events using LRS, we will focus on how they consider constitutive and alternative terminal ends.

**Table 1.** Computational approaches to identify and quantify mRNA terminal ends in LRS data

Approach	Known annotations	De novo identification	Terminal end selection	Orthogonal features	Full-length status	Reference
TAPIS	Optional	Clustering	Longest isoform with shared splice sites	Terminal end A's, terminal end adapters	No	(Abdel-Ghany et al. 2016)
StringTie2	Optional	Graph-based	Unknown	No	No	(Kovaka et al. 2019)
FLAIR	Yes	Clustering	Read density	Terminal end adapters	Read level	(Tang et al. 2020)
TALON	Yes	Clustering	Clustering	Terminal end A's	Transcript level, FSM/ISM/NIC categories	(Wyman et al. 2020)
FLAMES	Yes	Clustering	Annotations	SRS RNA-seq	No	(Tian et al. 2021)
Bambu	Yes	Probabilistic ML model	Categorization and modeling	Terminal end A's	Read level and transcript level	(Chen et al. 2023)
ESPRESSO	Yes	Clustering	Splice junctions of terminal exons	No	Read level and transcript level with FSM/ISM/NIC categories	(Gao et al. 2023)
IsoQuant	Optional	Graph-based	Clustering	Terminal end A's	No	(Prjibelski et al. 2023)
IsoTools	Yes	Graph-based	Peak calling, read density	No	No	(Lienhard et al. 2023)
SQANTI3	Recommended	Probabilistic ML model	Categorization and modeling	Terminal end A's, terminal end adapters, SRS RNA-seq, complementary SRS data sets	Transcript level, FSM/ISM/NIC categories	(Pardo-Palacios et al. 2024a)

Overall, computational analyses have shown that LRS data can improve the identification of new isoforms and new terminal ends, especially in poorly annotated species. For instance, Iso-Seq data from stickleback fish found that the identified isoforms began 99 nt upstream of previously annotated TSSs and ended 500 nt downstream from annotated PASs, on average (Naftaly et al. 2021). More broadly, high-confidence 5′ and 3′ terminal sites identified from LRS have increased downstream or upstream LRS read coverage, respectively (Naftaly et al. 2021; Kainth et al. 2023; Pardo-Palacios et al. 2024a). This is unlikely if most of these sites were derived from random truncation events, providing confidence in the identification and quantification of terminal ends from LRS. Therefore, the challenge for computational analyses of LRS data is to filter out spurious reads or sites and determine which novel terminal ends should be considered high confidence.

### Comparing to reference transcriptomes

One underlying assumption of these analyses is that most genes and isoforms in a cell should already have been discovered and, thus, contained in a reference transcriptome (Box 2; Tang et al. 2020; Wyman et al. 2020; Tian et al. 2021; Chen et al. 2023; Lienhard et al. 2023; Pardo-Palacios et al. 2024a). Therefore, the default mode for most methods is to intrinsically rely on reference transcriptomes and annotation databases to map and/or define transcripts from annotated genes. Thus, the most conservative analyses of LRS data directly compare groups of reads based on their alignment to previously annotated isoforms and choose terminal sites within read classes based on splicing patterns of known isoforms (Abdel-Ghany et al. 2016; Tang et al. 2020). High-confidence isoforms are those that share all splice sites with a known isoform and have terminal ends within 100 nt of the annotated isoform ends and/or the 5′ read ends within the first exon of the annotated gene (Abdel-Ghany et al. 2016; Kovaka et al. 2019). However, because reference databases often do not contain all alternative terminal sites, using databases to infer true terminal ends can lead to errors. For instance, isoforms identified by tools that use annotations to correct ends were less likely to overlap with those identified by orthogonal experimental approaches that directly identify terminal ends (Pardo-Palacios et al. 2024b).

Box 2. **Defining the ground truth for full-length transcript quantification and discovery**

When developing and evaluating new LRS sequencing approaches or analytical frameworks, it is critical to objectively assess the accuracy of the insights gained from the data. For terminal end identification and quantification, it is particularly crucial to assess whether the reads are full length. Several different biological features or synthetic constructs have been put forth as positive controls to define the ground truth for RNA terminal ends, but each of these options have strengths and limitations.

***Reference transcriptomes.*** Comprehensive reference annotations of known isoforms (including exon, intron, and junction connectivity annotations) are often used as the default ground truth with the assumptions that (1) most isoforms have already been observed and (2) isoforms in the database are validated (Tang et al. 2020; Wyman et al. 2020; Tian et al. 2021). Transcriptome annotations are easy to obtain and are well maintained for model organisms or commonly studied species, with the caveat that the maintenance or quality of the annotations varies across species. However, annotations may contain spurious isoforms that have only been observed once, may be incomplete (especially for lowly expressed or condition-specific isoforms), and/or may have inaccurate terminal ends. This can hamper novel isoform discovery, especially for machine learning models that may be trained to recognize features of ubiquitously expressed isoforms. Similarly, the precise estimation of terminal ends may be constrained by known isoform structures for methods that rely on annotated internal exon connectivity to select terminal ends.

***Single-exon genes.*** Genes with no known splice sites or alternative isoforms, estimated to be 2285 genes in the human genome (Jorquera et al. 2021), may provide an internal control for LRS analyses. Reads from these genes allow the assessment of how biases created during RNA extraction, library preparation, and sequencing influence terminal-end identification in situations in which no alternative events are present (Workman et al. 2019). However, their status as single-exon genes is again dependent on known annotations, which might be incorrect, and their use as a control set is conditional on sufficient expression levels within the data set. Finally, it is hard to train models on the limited number of single-exon genes (which often express shorter than average transcripts) (Movassat et al. 2019), and they are not useful for evaluating novel isoform discovery.

***Orthogonal experimental data sets.*** Direct experimental probing of 5′ and 3′ terminal ends can be used to complement LRS-based identification and quantification of terminal ends. One of the most readily available experimental approaches, SRS RNA-seq, can be used to increase confidence in RNA quantification by providing higher sequencing depth compared with that of LRS approaches. SRS data sets can be generated or downloaded easily, can provide higher statistical power for confidence in novel sites, and can feature well-established methods for correcting biases in these data sets. Thus, these data have been used to empirically determine a ground truth for splice junctions and terminal ends (Depledge et al. 2019; Kainth et al. 2023), which is of particular use for lowly expressed transcripts or splice junctions. However, SRS data sets often suffer from edge effects that limit the ability to directly identify and quantify terminal ends. Although computational approaches exist to overcome these biases (Carralot et al. 2012; Birol et al. 2015; Fiszbein et al. 2022), they do not provide a direct assessment of terminal ends.

Instead, alternative sequencing protocols can be used to directly enrich and characterize terminal ends. For instance, Cap Analysis of Gene Expression (CAGE) (Takahashi et al. 2012b), POINT-seq (Sousa-Luís et al. 2021), RAMPAGE (Batut and Gingeras 2013), and 5′ RACE (Scotto-Lavino et al. 2006) are common approaches to do targeted sequencing of 5′ ends, whereas QuantSeq, 3′READS, and 3pSeq are common approaches for 3′ end sequencing. In addition, because TSSs are known to fall within open chromatin regions, assay for transposase-accessible chromatin (ATAC-seq) data can be used as an orthogonal confirmation for 5′ ends (Pardo-Palacios et al. 2024a). Data from these methods set a ground truth to evaluate the accuracy of terminal ends derived from LRS reads or consensus isoforms (Pardo-Palacios et al. 2024b). Additionally, these orthogonal data can be used to filter out reads whose terminal ends are not consistent with empirically derived sites (Anvar et al. 2018; Alfonso-Gonzalez et al. 2023; Calvo-Roitberg et al. 2024). However, care is required when using these data sets, because the results are often tissue- or context-specific and thus need to be collected for every cell type or condition being analyzed. Furthermore, there are several reports of spurious or biologically unclear CAGE peaks within introns and last exons from 5′ cap pulldown methods, potentially owing to post-transcriptional cleavage events (Mercer et al. 2010, 2011; Malka et al. 2017; Haberman et al. 2023). These may not reflect true 5′ ends of steady-state mRNAs and thus can lead to the inclusion of truncated reads as false positives.

***Synthetic RNA pools.*** To evaluate technical biases introduced during library preparation and sequencing, it has become common to “spike-in” pools of synthetic RNA molecules with known sequences and defined structures. The two most commonly used sets are the External RNA Controls Consortium (ERCC) mix (Pine et al. 2016; Movassat et al. 2019) and the Spike-in RNA Variants (SIRV) (Paul et al. 2016) libraries, which include hundreds of molecules with variable, but known sequences, lengths, and expression levels. SIRV pools include molecules with isoform diversity, and one SIRV pool also contains extremely long (up to 13 kb) monoexonic molecules. Thus, any diversity in read length, sequence (i.e., SNPs), splice sites, or terminal sites in LRS reads can confidently be ascribed to technical biases or sequencing errors. However, these synthetic molecules do not reflect the true biological diversity of sequence or isoform usage and thus limit the ability to assess the accuracy of novel isoform discovery.

***Simulated data sets.*** In silico simulated reads data sets offer customizable ground-truth data sets that can incorporate known transcript structures, variable molecule lengths, high-complexity libraries, and a range of known expression levels. Furthermore, simulations can explicitly model truncated reads or other errors during sequencing. Because every step of simulated read generation is computationally programmed and the true value of each parameter can be stored, simulations represent the ultimate ground truth. Tools like Trans-NanoSim (Hafezqorani et al. 2020), IsoSeqSim (https://github.com/yunhaowang/IsoSeqSim [accessed July 24, 2024]), and PBSIM3 (Ono et al. 2022) can be used to simulate long reads from a reference transcriptome. SQANTI-SIM (Mestre-Tomás et al. 2023) can also simulate novel transcripts and orthogonal data (e.g., corresponding CAGE peaks). Finally, DeepSimulator (Li et al. 2020) can generate both reads and nanopore raw electrical signals. However, simulations can only be designed to incorporate known biases with clear expected outcomes or distributions and thus are limited by previous knowledge of technical and biological biases.

### De novo isoform discovery

Most methods also provide an option to conduct de novo isoform identification to discover novel isoforms. These approaches generally fall into three broad categories: clustering reads, implementing graph theory, and probabilistic modeling. The first category involves generating de novo transcript models by self-aligning or clustering LRS reads and merging them to generate one transcript model per cluster (Tilgner et al. 2014; Gordon et al. 2015; Wang et al. 2016; Komor et al. 2017; Tang et al. 2020; Wyman et al. 2020; Gao et al. 2023). This clustering is often done based on the consistency of internal splice sites, in which each read is compared to previously analyzed reads on the basis of splice junctions (Tang et al. 2020; Wyman et al. 2020; Gao et al. 2023). The second category of de novo isoform discovery approaches leverages graph theory, such that isoforms are determined by charting possible paths through splice sites or introns (using inexact matching algorithms to account for splice site shifts) (Kovaka et al. 2019; Prjibelski et al. 2023). For both the clustering and graph theory approaches, terminal sites are determined either by choosing the most represented site in the transcript cluster (Tang et al. 2020; Wyman et al. 2020) or by specifically clustering read starts and ends and then choosing the most-represented site or most likely site based on annotations (Kovaka et al. 2019; Prjibelski et al. 2023).

The third category of de novo isoform discovery methods involves probabilistic modeling to identify and classify high-confidence and/or full-length isoforms (Chen et al. 2023; Pardo-Palacios et al. 2024a). These approaches train machine learning models on read QC metrics and define a set of parameters by which to correct and filter reads. These parameters can then be used to assign confidence to transcripts and/or identify artefactual transcripts (Chen et al. 2023; Pardo-Palacios et al. 2024a). These models are trained on annotations, simulations, or curated LRS data sets (see Box 2), but in the absence of such data, they can be initiated with user-defined criteria. To derive a set of parameters, the models use sequence (e.g., genome sequence indicative of mispriming events, poly(A) density at the 3′ end, adapter sequences), coverage (e.g., isoform expression, variability in 5′ and 3′ site support), splice site information (e.g., known junctions, splice sites), and/or orthogonal data sets (e.g., SRS RNA-seq, experimental methods to identify 5′ and 3′ sites) to identify transcripts, determine statistical error rates, and quantify isoform expression levels after read or transcript filtering (Chen et al. 2023; Pardo-Palacios et al. 2024a). An advantage of these models is the ability to calculate the probability with which each read is likely to match a complete transcript and whether it can be uniquely assigned to a single transcript, which then allows for probabilistic estimation of transcript abundance using standard expectation maximization algorithms (Chen et al. 2023). The learning of informative parameters from 5′ and 3′ features enables these models to classify reads and transcripts by their full-length status.

### Leveraging orthogonal data sources

The integration of LRS and SRS data sets can increase confidence by leveraging exon connectivity and increased coverage provided by each method, respectively (Kainth et al. 2023). This is helpful not only for gene or transcript abundance estimates but also for creating putative transcript databases that support the discovery of novel splice sites or terminal ends (Depledge et al. 2019; Fiszbein et al. 2022; Kainth et al. 2023; Calvo-Roitberg et al. 2024; Pardo-Palacios et al. 2024a). For instance, SQANTI3 recently introduced a “TSS ratio” metric, which calculates the ratio of SRS read coverage downstream from TSSs derived from LRS reads relative to the upstream coverage, where true TSSs are expected to be depleted of upstream reads. This feature can be used to filter putative spurious TSSs and can be a feature in the downstream machine learning algorithm. Furthermore, SRS data from orthogonal experimental methods (e.g., CAGE [Takahashi et al. 2012a], ATAC-seq [Buenrostro et al. 2013], 5′ RAMPAGE [Takahashi et al. 2012a; Batut and Gingeras 2013], etc., for 5′ ends and Quant-seq [Moll et al. 2014], 3p-seq [Jan et al. 2011], etc., for 3′ ends) (see Box 2) can be also be used to fine-tune or filter terminal ends derived from LRS data. Although most approaches validate their isoform identification pipelines by comparing with data from these methods (Wyman et al. 2020; Tian et al. 2021; Lienhard et al. 2023; Pardo-Palacios et al. 2024a), some LRS approaches now allow for integrated analyses of these data sets to provide confidence for novel terminal end discovery. SQANTI3 can process complementary data sets from CAGE or Quant-seq experiments or annotations from the PolyASite database (Buenrostro et al. 2013; Herrmann et al. 2019) and reports the distance between terminal regions supported by empirical evidence and LRS terminal sites (Pardo-Palacios et al. 2024a).

More directly, several computational approaches take advantage of the sequence features intrinsic to terminal ends or terminal end adapters added during library preparation as outlined above. At the 3′ end, reads with nontemplated poly(A) tails can be used to cluster read ends and identify high-density sites as bona fide 3′ ends (Abdel-Ghany et al. 2016; Celik and Mortazavi 2022; Chen et al. 2023; Prjibelski et al. 2023; Pardo-Palacios et al. 2024a). In contrast, the enrichment of templated adenines or thymines in the first or last 20 nt of a read or a cluster of reads may indicate internal mispriming events and can be used to discount those sites as true 3′ ends (Wyman et al. 2020; Chen et al. 2023). Finally, several approaches identify 5′ or 3′ primers added during library preparation (either by ligation or during RT steps) to provide confidence in the identification of terminal sites from reads or consensus transcripts (Abdel-Ghany et al. 2016; Tian et al. 2021; Chen et al. 2023; Pardo-Palacios et al. 2024a). This allows approaches to selectively filter those reads (Tian et al. 2021) or perform a dual analysis with or without those reads (Chen et al. 2023).

### Categorizing full-length vs. incomplete isoforms

An alternative way to consider LRS analyses is to retain all data and instead categorize reads or potential consensus isoforms based on their likelihood of being full length. This is primarily done by assigning each isoform to a “completeness” class based on some or all of the features listed above, often using machine learning models that curate 3′ and 5′ descriptors to fine-tune classifiers (Wyman et al. 2020; Pardo-Palacios et al. 2024a). Transcripts with splice junctions that perfectly match known isoform models (generally based on annotations) are termed “full splice matches” (FSMs), with some flexibility at the 5′ and 3′ ends (FSM alt 5′ end or FSM alt 3′ end). Transcripts that overlap some junctions of an existing isoform are termed “incomplete splice matches” (ISMs), with flexibility at the 5′ and/or 3′ end or intron inclusion (ISM 5′ fragment, ISM 3′ fragment, ISM internal fragment, or ISM intron retention). Transcripts with no junctions are termed “monoexon,” and those with novel junctions or splice sites are termed “novel-in-catalog” (NIC) or “novel not-in-catalog” (NNC), respectively (where “in-catalog” refers to annotated sites). These categories provide information about potentially incomplete isoforms without rigid filtering and flexibility to decide which isoforms to include in downstream analyses. Approaches that use probabilistic modeling can also calculate the likelihood of individual reads matching complete isoforms (based on annotations) and, thus, provide two abundance estimates per isoform: one from reads that are likely to be full length and another that is more inclusive of reads that may be incomplete (Chen et al. 2023). This approach found that abundance estimates were more reproducible when only using reads that are likely to be full length (Chen et al. 2023). This framework also allows for analyses that require individual read information, such as those that investigate coordinated site usage across individual molecules.

### What's next? Remaining challenges for characterizing mRNA terminal ends with LRS technologies

Although LRS continues to offer new possibilities for deeper characterization of essential biological and genomic processes, it is important to be cautious when selecting the specific application and interpreting data, as technical and analytical biases may affect the results and/or lead to misleading findings. This risk is especially prevalent for the discovery or quantification of the ends of mRNA transcripts. As outlined above, LRS reads are likely to delineate spurious RNA terminal ends owing to numerous biases or issues introduced during the library preparation or sequencing stages. Despite advances in experimental and computational approaches to overcome these challenges, several key developments are needed for improved LRS-based discovery and characterization of mRNA terminal ends.

Many experimental steps during the generation of LRS libraries can be optimized, starting from the input material. Preservation of full-length transcripts is critical; therefore, care is needed during RNA extraction and library preparation to avoid shearing effects by pipetting too quickly or mixing reagents too aggressively. As mentioned above, during library preparation, several steps are potentially problematic; thus, further optimization of RT and the subsequent PCR amplification is needed to ensure the complete processing of the entire molecule and avoid the introduction of length biases, respectively. Increasing adapter lengths or more widespread use of UMIs at both ends of the molecule might help to resolve technical artifacts (Wyman et al. 2020; Ibrahim et al. 2021). Finally, although several approaches have been described to select and enrich full-length RNA molecules, further development is anticipated to enhance the robustness and throughput of these applications. One area of particular interest is the development of methods to directly identify 5′ end mRNA modifications (e.g., 5′ 7-methyl-guanosine caps) or sequences during direct RNA sequencing, which would likely require advances in both biological insight and technical capabilities (for reviews, see Kong et al. 2023; van Dijk et al. 2023).

Following library preparation, LRS sequencing instruments themselves require additional design considerations, particularly for ONT flow cells and/or instruments. More accurate detection methods for signal spikes or aberrant pauses can reduce error rates and improve basecalling accuracy. ONT can also improve the pore characteristics and control translocation specific for RNA applications, including the development of solid-state pores with more consistent characteristics and performance (Fragasso et al. 2020; Fried et al. 2021), appropriate translocation speed, and software ability to hold a strand within the pore and read the strand several times, which may allow both time for more accurate basecalling and repeated sensing of the terminal ends to improve coverage and increase confidence in basecalls. Additional robust methods are needed to address the challenges of accurate basecalling of the terminal 10–15 nt passing through the ONT pore, as well as continued basecalling following a spike in electrical current. Both PacBio and ONT continue to develop updated methods to boost throughput to increase coverage, which will allow higher basecalling accuracy (Jain et al. 2022; Al'Khafaji et al. 2024).

Finally, there is still room for development and optimization of LRS analyses approaches. Despite progress in the development of methods to correct or model biases, accurately identifying terminal ends remains an unsolved problem. Although filtering or classifying reads can improve confidence in terminal-end observations, these steps come at the cost of read coverage. Moreover, the reliance on external (often not sample-matched) data sets reduces the ability to confidently make novel observations. The current tools for isoform discovery generally involve either clustering, graph theory, and probabilistic modeling, and they estimate confidence using either reference annotations or orthogonal data sets. An inherent challenge across all of these analytical tools is the definition of a comprehensive “ground-truth” data set for training data or validating findings. The establishment of these data would enable further development of data-informed thresholds for calling true isoforms to better understand the trade-offs between sensitivity and accuracy across these approaches (particularly for the detection of rare isoforms) (Chen et al. 2023). When specifically thinking about terminal ends, it appears that the widespread anchoring of isoform models on splicing junctions prevents current methods from identifying isoforms that only differ in their start or end coordinates. Explicit modeling of technical processes that cause read truncation and incorporation of isoform-specific transcription and/or degradation rates may allow methods to overcome these limitations (Chen et al. 2023). Finally, there is a need for additional characterization of the statistical power for isoform discovery and quantification afforded across different LRS sequencing depths to better understand how many LRS reads are needed for different biological analyses.

Recent benchmarking studies have made significant strides toward standardizing depth and read length expectations in the field (Chen et al. 2021; Dong et al. 2023; Pardo-Palacios et al. 2024b), as well as evaluating both library preparation strategies and computational pipelines for transcript/gene discovery and quantification. The results of these studies have generally shown that PacBio delivers higher accuracy and longer reads compared with ONT, yet the cost per read is significantly lower for ONT (Mikheenko et al. 2022; Pardo-Palacios et al. 2024b). Additionally, the accuracy of both technologies is still lower than that for SRS (Pardo-Palacios et al. 2024b). Thus, the choice of which technology likely varies depended on specific research objectives and practical considerations. For projects aiming to quantify known RNA isoforms with high-throughput, the affordability of ONT technologies might be preferable. Conversely, the accuracy and longer reads provided by PacBio might be better suited for research focused on discovering novel isoforms or quantifying alternative terminal site usage. Importantly, these benchmarking studies continue to standardize assessment methods to ensure comparability in results across studies, identify systematic technical and computational challenges that must be addressed, and provide practical guidance for researchers in this field. However, rapid developments in LRS technology, both biologically and computationally, raise the need for caution when considering the conclusions of such benchmarking papers, as changes in chemistry and software versions can quickly make comparisons obsolete. Thus, continuous efforts are needed to ensure rapid assessments of these updates.

## Supplemental Material

Supplement 1

Supplement 2

Supplement 3
