# Supplementary material for: Challenges in identifying mRNA transcript starts and ends from long-read sequencing data
Source: Genome Res. 2024 Nov;34(11):1719–34. doi: 10.1101/gr.279559.124 (PMC11610588; doi:10.1101/gr.279559.124)
Supplement: Supplement 1 [file Supplemental_Methods.pdf]

Supplemental Methods

Sequence data sources

For all analyses, we used publicly available samples from A549, Hct116, HepG2, K562, MCF-7 cell lines. FASTQ files from Oxford Nanopore direct-cDNA and directRNA reads were downloaded from the Singapore Nanopore-Expression Project (SG-NEx) website (https://github.com/GoekeLab/sg-nex-data) (Chen et al. 2021). FASTQ files from Pacific Biosciences Iso-Seq reads were downloaded from the ENCODE Project data portal (https://www.encodeproject.org/) (Luo et al. 2020). The specific samples and accession numbers can be found in Table S1.

Long-read data mapping and processing

All long-read sequencing data were mapped to the hg38 human reference genome (Howe et al. 2021) using minimap2 (Li 2021) following the developers' recommendations. Specifically, for each sequencing type, the -ax option was modified as follows: *-ax splice* for directcDNA, *-ax splice -uf -k14* for directRNA, *-ax splice:hq -uf* for Iso-Seq. To assign reads to genes, primary alignments were split into individual features using *BEDtools bamtobed* and then *BEDtools intersect* was used to intersect the features with annotated genes. Only the reads where the start and end features were assigned to the first exon (FE) and poly(A) peaks for the same gene, respectively, were considered and used in the analysis of the terminal site. The length of the primary aligned reads was calculated from the BAM files. Counts per million were calculated by dividing the count of each gene by the total counts in the sample and multiplying by one million.

Terminal end classification and filtering

We used databases of empirically derived 5' end start sites or 3' end poly(A) sites to assess the accuracy of terminal ends in LRS reads. Specifically, we used two 5' start site annotations: (a) human CAGE peaks, downloaded from the FANTOM project website (Lizio et al. 2019) and (b) human HITindex first exons (Fiszbein et al. 2022). HITindex first exons were obtained by running the HITindex pipeline on short-read RNA-seq data from the entire GTEx V8 database (Carithers et al. 2015) and retrieving exons identified as first exons or hybrid first-internal exons in at least one tissue. For 3' end annotations, we used experimentally verified human

polyadenylation sites from the PolyASite 2.0 database (Herrmann et al. 2019), a consolidated atlas of polyadenylation sites from publicly available 3’ end sequencing datasets.

LRS reads were classified by their overlap with 5’ end start sites and/or 3’ end poly(A) sites from the sources above. For start sites, we intersected the upstream most feature of each read with either CAGE peaks or HITindex first exons using *BEDtools intersect* and considered overlap as read features that overlapped within +/- 75nt from the CAGE peak or exon 5’ and 3’ ends. Similarly, for end sites, we used *BEDtools intersect* to intersect the downstream most feature of each read with poly(A) peaks from the PolyASite database and retained reads whose ends overlapped with a region at least +/- 50nt of the peak.

Software availability

The script used to calculate truncation metrics and reproduce data figures is included as Supplemental Code and can be found at https://github.com/ezecalvo/tss_tes_terminal_truncation.

Supplementary References

Carithers LJ, Ardlie K, Barcus M, Branton PA, Britton A, Buia SA, Compton CC, DeLuca DS, Peter-Demchok J, Gelfand ET, et al. 2015. A Novel Approach to High-Quality Postmortem Tissue Procurement: The GTEx Project. *Biopreserv Biobank* **13**: 311–319.

Chen Y, Davidson NM, Wan YK, Patel H, Yao F, Low HM, Hendra C, Watten L, Sim A, Sawyer C, et al. 2021. A systematic benchmark of Nanopore long read RNA sequencing for transcript level analysis in human cell lines. *bioRxiv* 2021.04.21.440736. https://www.biorxiv.org/content/10.1101/2021.04.21.440736v1 (Accessed July 7, 2024).

Fiszbein A, McGurk M, Calvo-Roitberg E, Kim G, Burge CB, Pai AA. 2022. Widespread occurrence of hybrid internal-terminal exons in human transcriptomes. *Sci Adv* **8**: eabk1752.

Herrmann CJ, Schmidt R, Kanitz A, Artimo P, Gruber AJ, Zavolan M. 2019. PolyASite 2.0: a consolidated atlas of polyadenylation sites from 3′ end sequencing. *Nucleic Acids Res* **48**: D174–D179.

Howe KL, Achuthan P, Allen J, Allen J, Alvarez-Jarreta J, Amode MR, Armean IM, Azov AG, Bennett R, Bhai J, et al. 2021. Ensembl 2021. *Nucleic Acids Res* **49**: D884–D891.

Li H. 2021. New strategies to improve minimap2 alignment accuracy. *Bioinformatics* **37**: 4572–4574.

Lizio M, Abugessaisa I, Noguchi S, Kondo A, Hasegawa A, Hon CC, de Hoon M, Severin J, Oki S, Hayashizaki Y, et al. 2019. Update of the FANTOM web resource: expansion to provide

additional transcriptome atlases. *Nucleic Acids Res* **47**: D752–D758.

Luo Y, Hitz BC, Gabdank I, Hilton JA, Kagda MS, Lam B, Myers Z, Sud P, Jou J, Lin K, et al. 2020. New developments on the Encyclopedia of DNA Elements (ENCODE) data portal. *Nucleic Acids Res* **48**: D882–D889.
